# Leaf Morphological and Epidermal Traits Variability along an Environmental Gradients in Ten Natural Populations of *Pistacia lentiscus*

**DOI:** 10.3390/life13071617

**Published:** 2023-07-24

**Authors:** Abdelghafour Doghbage, Safia Belhadj, Fathi Abdellatif Belhouadjeb, Hassen Boukerker, Jean Philippe Mevy, Thierry Gauquelin, Alain Tonetto, Saifi Merdas, Bakria Touati, Fethi Saimi, Rafik Rahem, Arezki Derridj, Feriel Foulla Hassen, Walid Soufan

**Affiliations:** 1 Faculté des Sciences Biologiques et Agronomiques, Université Mouloud Mammeri de Tizi-Ouzou, Tizi Ouzou 15000, Algeria; aderridj@yahoo.fr; 2 Centre de Recherche en Agropastoralisme (CRAPAST), Djelfa 17000, Algeria; belhouadjebfathi@gmail.com (F.A.B.); agrotitou@live.fr (B.T.); fethisam2@gmail.com (F.S.); rafikk06@gmail.com (R.R.); 3 Département Agro-Vétérinaire, Faculté des Sciences de la Nature et de la Vie, Université Ziane Achour de Djelfa, Djelfa 17000, Algeria; belhadjsafia@yahoo.fr (S.B.);; 4 Scientific and Technical Research Center on Arid Regions, Biskra 07000, Algeria; boukerker.hassen@crstra.dz (H.B.); saifieco@gmail.com (S.M.); 5 Aix Marseille Université, Avignon Université, Centre National de la Recherche Scientifique (CNRS), Institut de Recherche pour le Développement (IRD), Institut Méditerranéen de Biodiversité et d’Ecologie Marine et Continentale (IMBE), 13397 Marseille, France; jean-philippe.mevy@imbe.fr (J.P.M.); thierry.gauquelin@imbe.fr (T.G.); 6 Aix Marseille Université, CNRS, Centrale Marseille, Fédération Sciences Chimiques Marseille (FSCM), Plateforme de Recherche Analytique Technologique et Imagerie (PRATIM), 13397 Marseille, France; alain.tonetto@univ-amu.fr; 7 Centre de Recherche en Aménagement du Territoire (CRAT), Campus Zouaghi Slimane, Route de Ain El Bey, Constantine 25000, Algeria; 8 Plant Production Department, College of Food and Agriculture Sciences, King Saud University, P.O. Box 2460, Riyadh 11451, Saudi Arabia

**Keywords:** macromorphology, ultrastructure, aridity, altitude, leaf, stomata, trichome, xeromorphic

## Abstract

The species belonging to the genus *Pistacia* possess ecological, economic, and medicinal value. They show a very high ecological plasticity. This research is a contribution to the study of the intraspecific diversity and variability of 10 populations of *Pistacia lentiscus* in different bioclimates. Nine locations in Algeria and one site in France have been selected in order to understand the strategies developed by this species under extreme conditions, including altitude and aridity, and to identify the adaptive processes that can be observed based on the morphological and ultrastructural features of the leaf. As a result of this research, we have collected a large quantity of important information on morphological and microphytodermal leaf variability for the ten studied populations. The statistical analyses showed a very important difference in the studied characteristics between these populations. It has been demonstrated that environmental factors also have a significant impact on the heterogeneity of most measured leaf features. Moreover, the observations with the scanning electron microscope (SEM) enabled us to highlight new characteristics of the studied species, such as the glandular trichomes on the leaflets and embedded stomata in the epidermis. These criteria could supplement the existing morphological characteristics used in the systematic classification of the *Pistacia* genus. Overall, the studied species have shown xeromorphy features, which give them the opportunity to be used in desertification mitigation programs, due to their ability to withstand conditions of extreme aridity.

## 1. Introduction

The sustainable development of an ecosystem is based above all on the rational management of its natural resources: soil, water, and vegetation. However, forest species are part of the natural resources that we must protect and make better use of. Unfortunately, there probably no longer exists any ecosystem that does not bear the imprint of human influence [1].

Individuals and populations are not passively influenced by environmental factors. They present varying degrees of ecological plasticity, allowing them to adapt to the temporal and/or spatial fluctuations of the limiting factors in the environments to which they are subservient by developing regulatory mechanisms which cause morphological and physiological modifications. These mechanisms allow them to maintain constant internal conditions at optimal values in the face of environmental variability [2]. In this context, reintroducing indigenous species adapted to climatic hazards such as *Pistacia lentiscus*, which is widely known and used by local populations, constitutes a sustainable way of rehabilitating degraded lands.

*P. lentiscus*, a dioecious shrub with evergreen alternate leaves from the typically Mediterranean Anacardiaceae family, is widely distributed in Algeria. This wide distribution area is due to the tolerance of species of this genus to adverse environmental conditions such as drought and poor soils, which makes them attractive for use in reforestation and sylviculture programs in arid and semi-arid areas. In addition to their ecological function, these species provide other economic and socio-cultural functions.

Systematically and ecologically, the genus *Pistacia* remains poorly defined, despite the contribution of several authors. These problems, raised in [3], were then addressed by taking into account macromorphological characteristics as well as micromorphological elements and more recently by using molecular markers [4]. The characteristics of the leaf epidermis have proven to be useful criteria to support taxonomic studies between *Pistacia* species. However, there are few studies on the leaf epidermis characteristics of the genus *Pistacia* using light and scanning electron microscopy (SEM). Nevertheless, we can cite the comparative study of the micromorphology of the leaf epidermis of eight populations of *Pistacia atlantica* from Algeria [5,6,7], the study of the morpho-anatomical variability and terpene composition contained in the leaves of *Pistacia atlantica* [8], and the study of foliar microphytodermal characterization of *Pistacia lentiscus* under different bioclimates [9]. It is in this context that our work fits, which constitutes a contribution to the study of the diversity and variability of 10 provenances of *P. lentiscus* sampled from various bioclimates. This study provides a better understanding of the strategies developed by this species in habitats with extreme conditions, in particular altitude and aridity, and the adaptive processes which it is likely to reveal, based on morphological and ultra-structural traits of the leaf, which is the plant organ most exposed to environmental conditions and evolutionary processes.

In addition, our study also aims to contribute to a better knowledge of species of the genus *Pistacia* and the possibility of integrating them into reforestation programs and the fight against desertification.

## 2. Materials and Methods

### 2.1. Sampling

**Figure 1:**
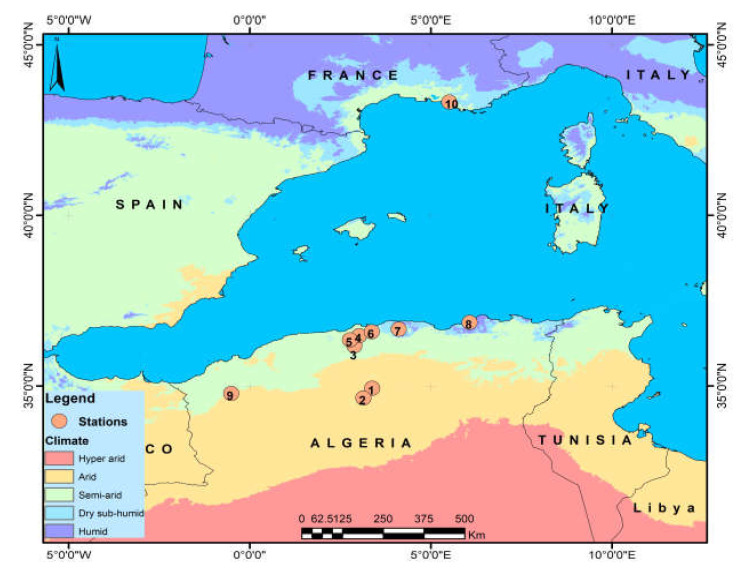
The location of stations by bioclimate (1: Dar), (2: Sb), (3: Berr), (4: Oma), (5: ElHam), (6: Boum), (7: Tiz), (8: Saz), (9: Tgh), (10: Marss).

Sampling for *Pistacia lentiscus* leaves was conducted across 7 regions in Algeria and 1 site in Marseille, France, covering diverse bioclimatic conditions. The selection of sites (9 in Algeria) followed a transect spanning from north to south and east to west, including varying altitudinal and aridity gradients. Additionally, one site was included in Marseille, France, resulting in a total of 10 sampling sites. (Table 1, Figure 1).

### 2.2. Morphological and Ultrastructural Study of Leaves

#### 2.2.1. Leaf Macro-Morphology

The leaves have been air-dried and stored in paper bags in the laboratory. The measurements concerning the quantitative and qualitative morphological parameters concerned all the individuals within each population. For this, we based ourselves on the repositories [10].

A total of 15 quantitative and qualitative characteristics were measured: (1) leaf length (Ll) (cm), (2) leaf width (Lw) (cm), (3) terminal leaflet length (TLl) (cm), (4) terminal leaflet width (TLw)(cm), (5) terminal leaflet length/width ratio (L/w TL), (6) petiole length (Pl) (cm), (7) number of leaflet pairs (Nl), (8) leaf rachis wing (Lrw), (9) leaf margin (Lm), (10) leaf color (Lc), (11) presence of terminal leaflet (TL), (12) size of terminal leaflet relative to basal leaflets (STl), (13) shape of the terminal leaflet (ShTl), (14) form of the apex of the terminal leaflet (FA), and (15) form of the petiole (FP) (Table A1).

#### 2.2.2. Epidermis, Stomata, and Trichomes

Light microscopy: the leaf impression technique was applied to view stomata. A thin layer of clear nail varnish was painted onto both adaxial and abaxial leaf surfaces and left for 5 to 10 min. A strip of transparent sticky tape (sellotape) was placed over the dried varnish and pressure was applied to obtain an imprint. The sellotape with its imprint was peeled from the leaflets and placed onto a glass microscope slide. Replicas were examined under an optical microscope (OPTICA AXIOM 7000). Pictures were digitally recorded for each slide at magnifications ×100 and ×400 and used for stomata measurements. The length and width of ten stomata per leaf were measured on the abaxial face. In addition, the stomata in ten adaxial and ten abaxial areas of five leaves per population were counted.

Scanning electron microscopy: The other leaves were cleared out with ethanol (90%) in order to remove external particles and dust, then standard procedures were followed using a Scanning Electron Microscope (SEM) to study the epidermal leaf surfaces. Three specimens from each site were examined. A section of 5 mm^2^ of the dry leaf surface (both adaxial and abaxial surfaces) was fixed on a labeled stub. The samples were coated with gold and scanned in a Philips XL 30 ESEM (Philips Electronic Instruments Co, Mahwah, NJ, USA). SEM pictures were digitally recorded in different magnifications.

Observations and measurements were made and related to thirteen quantitative and qualitative characteristics of stomata and trichomes, namely:

− Qualitative characteristics: shape, distribution and position of stomata on the epidermis, repartition and density of trichomes (leaf margins, adaxial and abaxial surfaces, along the veins), and types of trichomes.
− Quantitative characteristics: length and width of stomata on the abaxial face (µm) and stomatal density on both abaxial and adaxial faces (number of st/mm^2^).

### 2.3. Statistical Data Processing

The descriptive statistics, the correlation coefficient of the variables measured with the station parameters (altitude and aridity), the analysis of variance (ANOVA) was carried out in order to highlight the effect of provenance on the variability of the different measured parameters, followed by the Newman–Keuls test (HSD) to detect homogeneous groups.

Principal Component Analysis (PCA) has been carried out in order to highlight the spatial distribution of individuals (typology) according to their morphological and ultrastructural characteristics, and also to identify the markers that contribute to their discrimination. This analysis is performed using STATISTICA version 12 software.

## 3. Results

### 3.1. Inter-Population Variability of Quantitative Macro-Morphological Traits of the Leaf and Terminal Leaflet

#### 3.1.1. Provenance Effect on the Leaf

The lengths of *P. lentiscus* leaves observed across different sites displayed an average variation of 8.16 cm ± 1.73, ranging from a minimum of 3.2 cm ± 1.73 to a maximum of 14.5 cm ± 1.73. As for the width, recorded values ranged from 1.3 cm ± 1.14 to 10.2 cm ± 1.14, with an average width of 5.09 cm ± 1.14.

Regarding the leaflets, *P. lentiscus* typically possesses between 4 ± 1.96 and 16 ± 1.96 leaflets, with an average of 9.07 leaflets. The most frequently observed number of leaflets is 8 (28.71%). The length of the petiole varied between a minimum of 0.5 cm ± 0.41 and a maximum of 4 cm ± 0.41, with an average of 1.44 cm ± 0.41. The coefficient of variation for petiole length is equal to 28.32% (Table 2).

**Figure 2:**
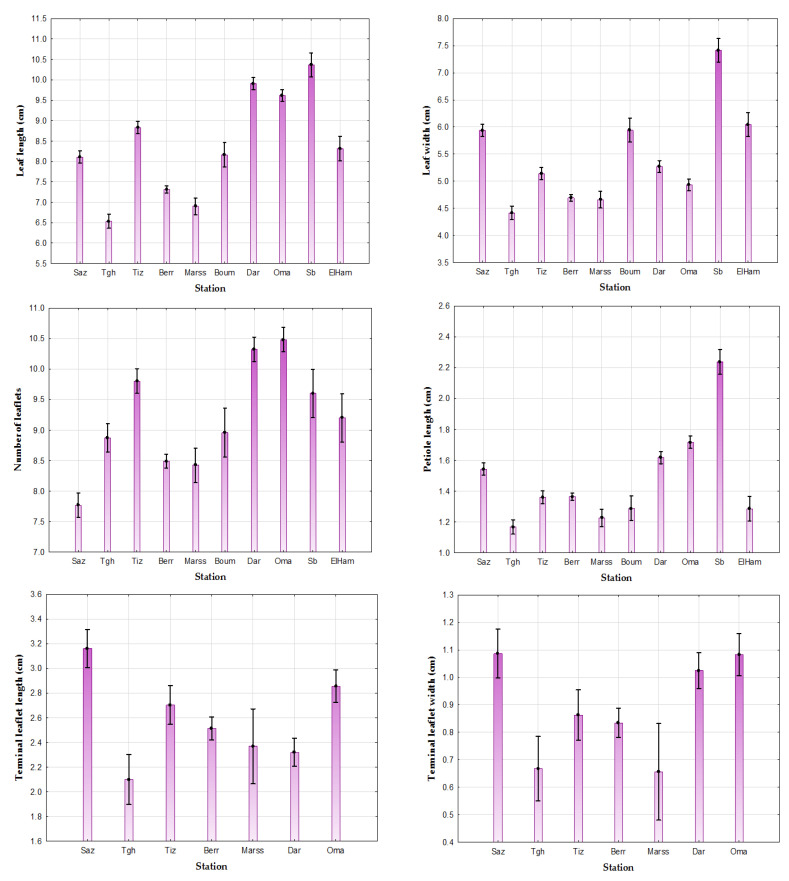

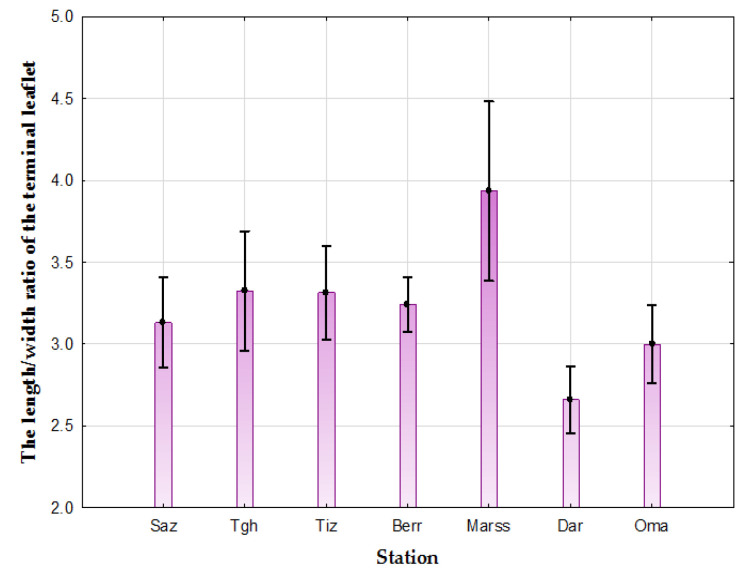
Provenance effect by ANOVA (α = 0.05) on leaf biometrics and TL in *Pistacia lentiscus*.

The leaves are longer in Senalba (Sb) (10.36 cm), Dar Chioukh (Dar) (9.9 cm), El Omaria (Oma) (9.61 cm), Tizi-Ouzou (Tiz) (8.82 cm), El Hamdania (ElHam) (8.31 cm), Boumerdès (Boum) (8.16 cm), and Jijel (Saz) (8.10 cm), and shorter in Tlegh (Tgh) (6.53 cm), Marseille (Marss) (6.9 cm), and Berrouaghia (Berr) (7.31 cm). On the other hand, they are wider in Sb (7.41 cm), ElHam (6.04 cm), Boum (5.94 cm), Saz (5.93 cm), Dar (5.27 cm), and Tiz (5.13 cm), and less wide at Tgh (4.41 cm), Marss (4.65 cm), and Berr (4.69 cm). The number of leaflets varies from one population to another, the highest average was recorded in Oma (10.48), Dar (10.32), Tiz (9.8), Sb (9.6), and Boum (8.96), while the smallest number of leaflets was observed at Saz (7.76), Marss (8.42), and Berr (8.49). The petiole is longer at Sb (2.23 cm), Oma (1.71 cm), Dar (1.61 cm), and Saz (1.54 cm); however, small values were recorded at (Tgh) (1.16 cm) and (Marss) (1.22 cm) (Table 2, Figure 2).

The ANOVA for leaf dimension (length/width), number of leaflets, and petiole length shows a significant difference at threshold α = 0.05 between populations of *P. lentiscus*, with the results obtained for leaf biometrics by the Newman–Keuls test at a risk α = 0.05 revealing the presence of:

- Seven distinct groups for the length of the leaf: Group 1: Saz, Boum, and ElHam; Group 2: Oma and Dar; Group 3: Tgh; Group 4: Marss; Group 5: Berr; Group 6: Tiz; and Group 7: Sb.
- Six distinct groups for the width of the leaf: Group 1: Saz, Boum, and ElHam; Group 2: Marss and Berr; Group 3: Oma and Tiz; Group 4: Tiz and Dar; Group 5: Tgh; and Group 6: Sb.
- Six distinct groups for the number of leaflets: Group 1: Marss, Berr, and Tgh; Group 2: Berr, Tgh, and Boum; Group 3: Tgh, Boum, and ElHam; Group 4: Sb and Tiz; Group 5: Dar and Oma; and Group 6: Saz.
- Six distinct groups for the length of the petiole: Group 1: ElHam, Boum, Tiz, and Berr; Group 2: ElHam, Boum, and Marss; Group 3: Marss and Tgh; Group 4: Saz and (Dar); Group 5: Oma; and Group 6: Sb (Table 3).

#### 3.1.2. Provenance Effect on the Terminal Leaflet

When it exists, the average value for the terminal leaflet length is equal to 2.58 ± 0.67 cm, ranging between a minimum of 0.6 ± 0.67 cm and a maximum of 4.81 ± 0.67 cm, and a coefficient of variation is 26.24%. For the width, the values recorded vary between 0.10 ± 0.38 cm and 2.6 ± 0.38 cm with an average of 0.92 ± 0.38 cm, and the coefficient of variation is equal to 41.02%. The length/width ratio averages 3.1 ± 1.14, with values between a minimum of 1.16 ± 1.14 and a maximum of 17 ± 1.14. The coefficient of variation is equal to 36.91% for this ratio (Table 2).

The variance analysis of (ANOVA) for the terminal leaflet dimensions and the length/width ratio reveals a significant difference at the threshold α = 0.05 between the different populations of *P. lentiscus* with *p* < 0.05 (Figure 2).

The results obtained for the terminal leaflet biometry by the multiple comparison of the means with the Newman–Keuls test at a risk α = 0.05 reveals the presence of:

- Five distinct groups for the length of the terminal leaflet: Group 1: Tgh, Dar, and Marss; Group 2: Dar, Marss, and Berr; Group 3: Berr and Tiz; Group 4: Tiz and Oma; and Group 5: Saz.
- Three groups for the width of the terminal leaflet: Group 1: Dar, Oma, and Saz; Group 2: Marss and Tgh; and Group 3: Berr and Tiz.
- Three groups for the length/width ratio of the terminal leaflet: Group 1: Oma, Saz, Berr, Tiz, and Tgh; Group 2: Dar, Oma, Saz, and Berr; and Group 3: Marss. (Table 2).

#### 3.1.3. Principal Component Analysis (PCA)

**Figure 3:**
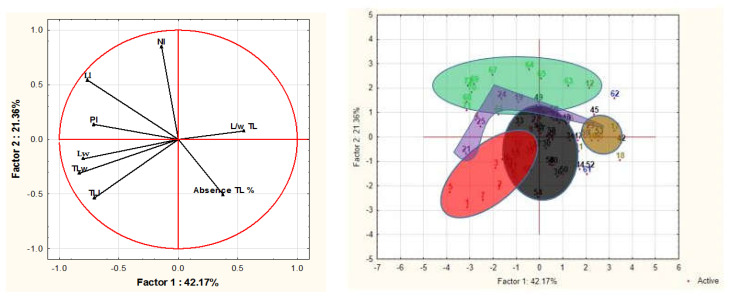
Circle of correlations and projection of individuals on factors 1 and 2.

The individuals of different populations of *Pistacia lentiscus* studied for the eight quantitative morphological characteristics were the subject of a principal component analysis (PCA). The interpretation of the results has been focused mainly on the first two axes (axes 1 and 2). The information provided by these two selected axes represents 63.52% of the total variance (Figure 3). To identify the variables responsible for the variances on the selected axes, we used their factorial weights (Table 3) which are the correlations of the variables with the factors. Only the first two factors (axes) present variables with factorial weights ≥0.6. It emerges that the majority of the variables measured are strongly negatively correlated on axis 1, while the number of leaflets is negatively correlated on axis 2 (Table 3).

Figure 3 shows the projections of the individuals analyzed in relation to axes 1 and 2. It can be clearly seen that the populations of *P. lentiscus* are well separated, which means that they are morphologically different by leaf characteristics. The characteristics (variables) responsible for this discrimination are those whose correlations with the axes concerned are high (≥0.7) (Table 3). Five distinct groups have been distinguished (Figure 3). This observation was confirmed by ANOVA, which highlights the existence of a significant difference between the different species from different bioclimates (Algeria and France). Group 1 (in green) represents the individuals of Dar, characterized by a high number of leaflets compared to the other populations, and by higher values for the length of the leaf and petiole.

Group 2 (in red) includes the Saz population, characterized by high dimensions (length and width) of the terminal leaflet as well as a higher leaf width compared to the other provenances. Group 3 (in purple) includes the individuals of Tiz, whose L/l ratio of TL, leaf width, and length of the petiole are very high. Group 4 (in khaki) is made up of individuals of Tgh, characterized by a very high L/l ratio of the TL, as well as for the rate of absence of leaflets. On the other hand, the dimensions (length and width) of the leaf and the TL as well as petiole length were shorter compared to the other populations studied. Group 5 (in black) includes the individuals of Berr whose leaf variables, TL, and petiole length are of intermediate dimensions (Figure 3).

### 3.2. Inter-Population Variability of Qualitative Macro-Morphological Traits of the Leaf and Terminal Leaflet

The majority of the leaves present foliar wings (wide) on the rachis to the petiole (97.82%). Almost half of the leaves have a dark green color (50.16%) and the other half have a green color (45.48%). The light green color represents 3.17%; other colors were recorded with a very low rate (1.18%). All of the leaves have full-margined leaflets (100%). Concerning the shape of the petiole, all the leaves have a rounded petiole on their lower face (100%) (Table 4).

A total of 18.49% of *P. lentiscus* leaves possess TF. The size of this leaflet compared to the basal leaflets is either smaller (11.18%), the same size (4.89%), or larger (2.52%). The shape of this most frequent leaflet is narrow elliptical with a rate of 17.74% (again in relation to the total number of leaves). The lanceolate and elliptical forms were also observed but with very low rates (0.43%, and 0.53%, respectively). For the apex, the mucronulated form is the most dominant (14.89%), other forms were recorded at low rates: obtuse (1.12%), acute (1.07%), cupsid (0.69%), and retuse (0.37%) (Table 4).

#### 3.2.1. Leaf

By provenance, all the leaves of *P. lentiscus* show the presence of leaf wings at the level of the rachis and the petiole with very high percentages, 98.99% (Saz), 82.43% (Tgh), 100% (Tiz, Berr, and Marss). The color of the leaves is variable in the different studied populations. Indeed, the dark green color is dominant in Saz (73.24%) and Berr (59.33%), on the other hand, it was not recorded in the population Tgh. The green color is more frequent in Tgh (99.54%), Marss (60%), and Tiz (58.47%), whereas the light green color was observed only in Saz, Tgh, and Berr but with very low occurrences (1%, 0.45%, and 6.11%, respectively) (Table 4).

#### 3.2.2. The Terminal Leaflet

By provenance, the absence of TL was reported at all stations with very high proportions: 78.93% (Saz), 82.88% (Tgh), 79.58% (Tiz), 81.33% (Berr), and 89.33% (Marss). The majority of provenances have smaller TFs compared to basal leaflets: Tgh (8.55%), Tiz (13.49%), Berr (13.44%), and Marss (6%). Meanwhile, the population Saz (8.36%) presents TL and basal of the same size. Regarding the shape of the TL, the narrow elliptical shape is the most frequent with variable rates: Saz (19.73%), Tgh (17.12%), Tiz (19.37%), Berr (17.56%), and Marss (9.33%). For the apex of this leaflet, the mucronulate form is the most widespread at all stations with different proportions: Saz (15.05%), Tgh (14.41%), Tiz (19.03%), Berr (14.66%), and Marss (8.66%). The acute, obtuse, cuspidate, acuminate, retuse (Berr and Tiz), caudate (Marss), and emarginate (Saz) forms were also recorded but at lower occurrences (Table 4).

### 3.3. Micromorphological Traits of the Leaf

#### 3.3.1. The Trichomes

**Figure 4:**
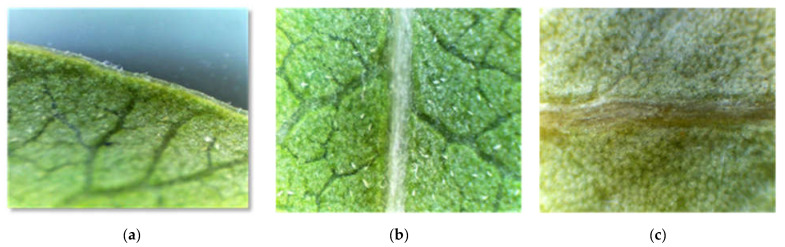
Leaf pilosity of different parts of the *Pistacia lentiscus* leaf under the binocular magnifying glass, (**a**): margin, (**b**): abaxial side, (**c**): major midrib.

**Figure 5:**
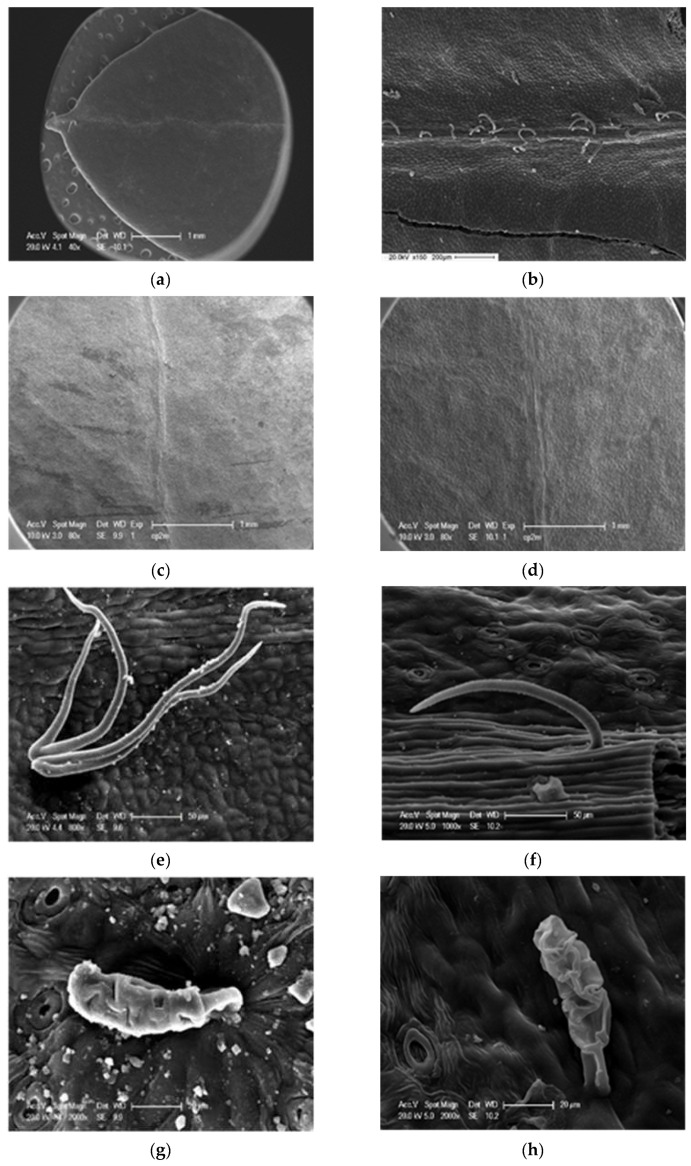
Micrograph (SEM) showing leaf trichomes of *P. lentiscus*, (**a**) margin (Dar), (**b**) principal nerve (principal drib) of adaxial side (Tiz), (**c**) adaxial side (Tgh), (**d**) abaxial side (Tgh), (**e**,**f**) non-glandular (ciliated) trichome, (**g**,**h**) glandular trichome.

In *P. lentiscus*, the majority of leaves are devoid of hairs at the margin (67.95%), on the central nerve (58.01%), on the upper side (60.32%), or on the lower side (54.94%), and when they are present, they are found with a low density (Table 4, Figure 4 and Figure 5).

For the different provenances, the leaves are characterized by the presence of cilia on the margin, but with very low densities: 89.63% (Saz), 77.02% (Tgh), 35.29% (Tiz), 5% (Berr), and 5.33% (Marss) (Table 4).

The presence of trichomes on the midrib (nerve) of the two upper and lower faces is low, especially at the level of the populations: Tiz (29.41%), Berr (27.66%), and Marss (8.66%). The same observation is true for the blades on both sides as very few leaves have hairs (Table 4). SEM photographs revealed the presence of glandular trichomes of the peltate type and non-glandular, cover, ciliate-type trichomes on both sides with a low density (Figure 5).

#### 3.3.2. The Stomata

**Figure 6:**
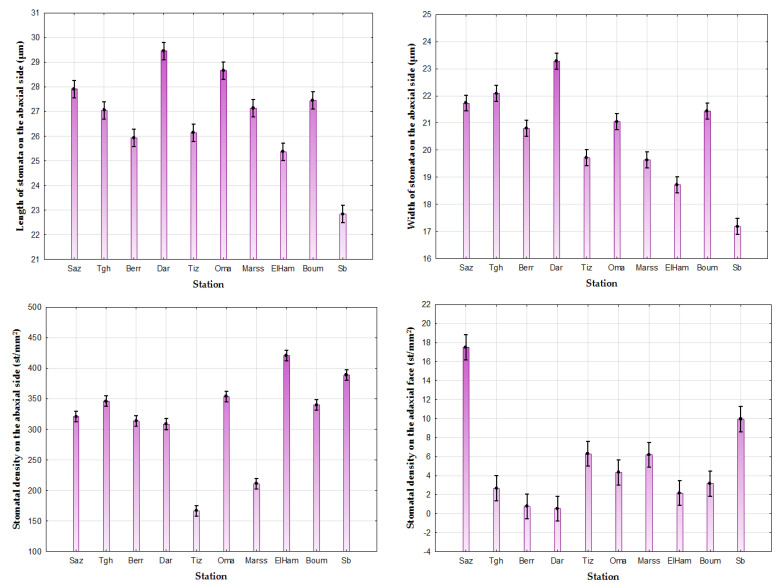
Provenance effect by ANOVA (α = 0.05) on abaxial and adaxial stomatal size and density of stomata in *Pistacia lentiscus*.

The values of the stomata length vary between 12.71 ± 3.38–42.67 ± 3.38 µm with an average of 26.88 ± 3.38 µm and a coefficient of variation equal to 12.60%, while the width values vary between 11.55 ± 2.96–37.74 ± 2.96 µm with an average of 20.71 ± 2.96 µm (Table 5). The results show that the greatest values of the length and the width of the stomata have been recorded at Dar (respectively, 29.46 ± 4.15 µm; 23.27 ± 2.76 µm), while the Sb station has the lowest values (22.84 ± 2.87µm; 17.18 ± 2.27 µm, respectively) (Table 5). ANOVA revealed a significant difference at the threshold α = 0.05 between the different populations with *p* < 0.05 (Figure 6). Similarly, the comparison of the means (α = 0.05) revealed the presence of seven groups for the length of stomata (Group 1: Tgh, Marss, and Boum, Group 2: Berr and Tiz, Group 3: Boum and Saz, Group 4: Sb, Group 5: ElHam, Group 6: Oma, and Group 7: Dar); eight distinct groups for stomatal width (Group 1: Marss and Tiz, Group 2: Berr and Oma, Group 3: Oma and Boum, Group 4: Saz and Boum, Group 5: Tgh and Saz, Group 6: Sb, Group 7: ElHam, and Group 8: Dar (Table 5).

**Figure 7:**
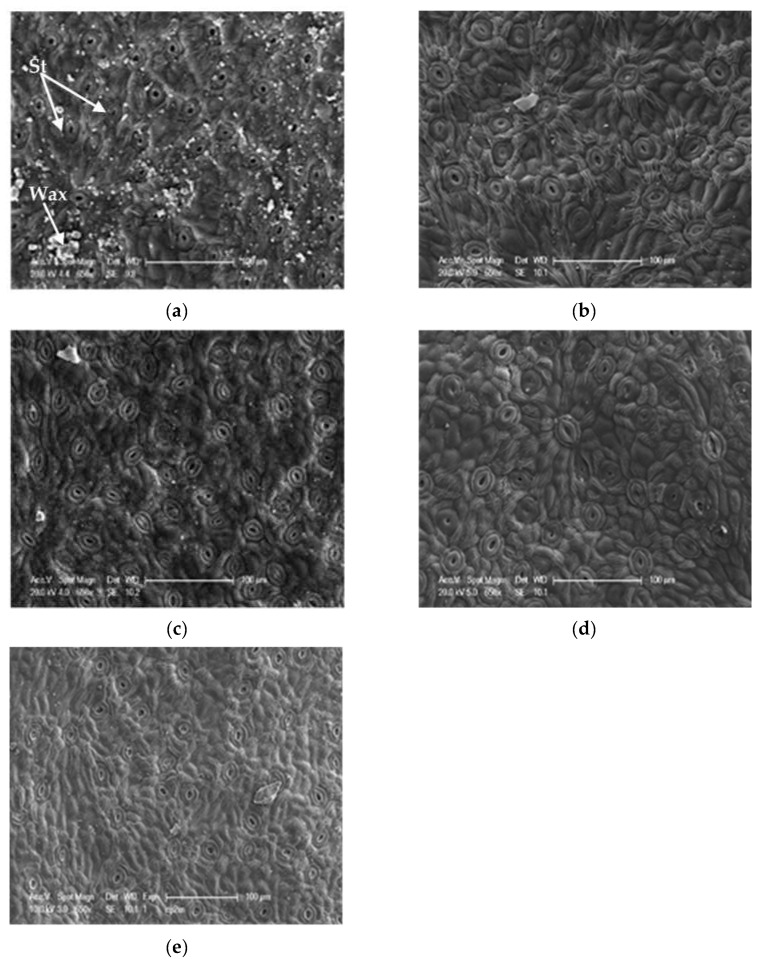
SEM photographs showing the shape, density, and position of stomata on the abaxial surface of the leaf epidermis of different populations of *P. lentiscus*, (**a**,**b**) Berrouaghia, (**c**) Taksept, (**d**) Sidi Abdelazziz, (**e**) Tlegh.

**Figure 8:**
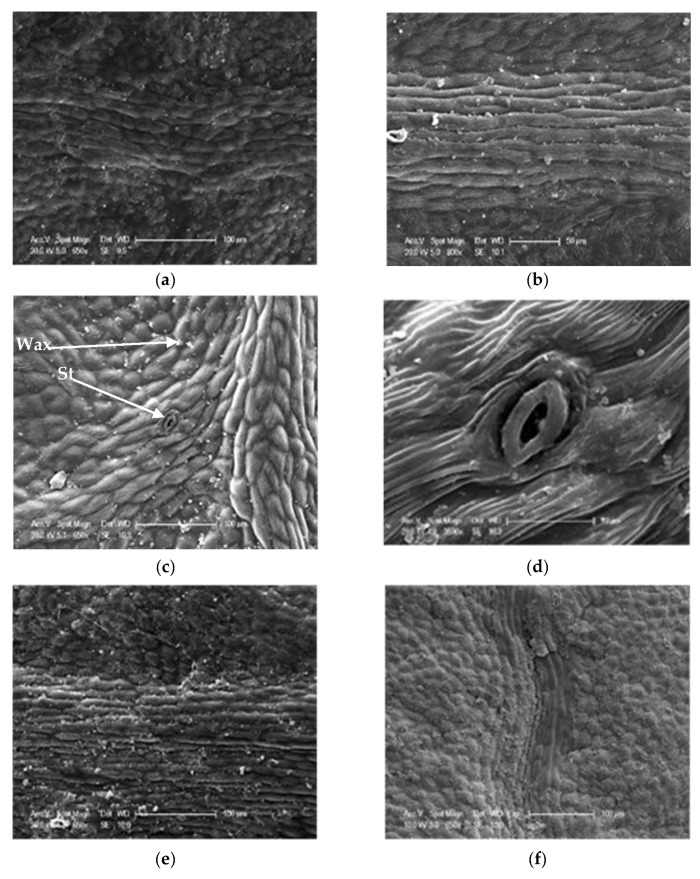
SEM photographs showing the density and position of stomata on the principal nerve (principal drib) of the leaf epidermis adaxial surface of *P. lentiscus* different populations, (**a**) Dar Chioukh, (**b**) Berrouaghia, (**c**,**d**) Taksept, (**e**), Sidi Abdelazziz, (**f**) Tlegh.

For stomatal density, the values vary between 75 and 600 st/mm^2^ on the abaxial side with an average of 319.05 st/mm^2^, while on the adaxial side of the leaf, where the number of stomata is less important, it swings between 0 and 125 st/mm^2^ with an average of 5.14 st/mm^2^ (Table 5). Our results show that the leaves have different densities in the 10 studied populations (Figure 7). They are higher on the abaxial side at ElHam (420 st/mm^2^) and Sb (388.6 st/mm^2^), on the other hand, the stations Tiz and Marss have the lowest values with 166.4 st/mm^2^ and 210.83 st/mm^2^, respectively. For the adaxial face, the few stomata were concentrated along the central nerve (central drib) (Figure 8). The highest values have been recorded at Saz (17.50 st/mm^2^) and Sb (9.93 st/mm^2^), while stations Dar (0.53 st/mm^2^) and BerrCh (0.76 st/mm^2^) have the lowest values (Table 5). A significant difference at the threshold α = 0.05 between the populations of *P. lentiscus* with *p* < 0.05 was recorded between the two abaxial and adaxial faces (Figure 6). The comparison of the means divides the stations into several groups: six distinct groups for stomatal density on the abaxial face (Group 1: Saz, Berr, and Dar, Group 2: Boum, Tgh, and Oma, Group 3: Tiz, Group 4: Marss, Group 5: Sb, and Group 6: ElHam); six distinct groups for stomatal density on the adaxial side (Group 1: Dar, Berr, ElHam, and Tgh, Group 2: Boum, Berr, ElHam, and Tgh, Group 3: Boum, Oma, ElHam, and Tgh, Group 4: Oma, Marss, and Tiz, Group 5: Sb, and Group 6: Saz) (Table 5).

## 4. Discussion

The present work is a contribution to the knowledge of the different species of the genus Pistacia, present in Algeria, through specific characteristics related to the leaves and stomata which can be used as identification criteria. This study will also allow us to understand the intra- and inter-population variations observed and the adaptive processes that they are likely to reveal. This last point seems essential to better address the diversity and variability of this species and its adaptation to the environments in which they evolve. According to [11], plants develop adaptive strategies in the face of the pressures of their environment in order to increase their tolerance interval and acquire an extended distribution area, through a set of morphological and physiological characteristics, which are the expression of their adaptation to the environment. According to [12,13], plants favor different functional traits in order to minimize the impact of drought. In light of the different results obtained, it is established that this species has xeromorphic characteristics which allow it to live in dry environments and better resist drought, which reflects its wide geographical distribution.

On the macromorphological level of the leaf, the results of quantitative traits obtained during our study show the existence of a great heterogeneity between the provenances for all the variables measured. Indeed, the leaves are longer in Sb (IA: 0.2; Altitude (Alt.) 1296 m), Dar (IA: 0.18; Alt. 1350 m), Oma (IA: 0.35; Alt. 779 m), Tiz (IA: 0.5; Alt. 209 m), Boum (IA: 0.45; Alt. 495 m), and Saz (IA: 0.65; Alt. 24 m), and shorter at Tgh (IA: 0.18; Alt. 963 m), Marss (IA: 0.43; Alt. 216 m), and Berr (IA: 0.39; Alt. 1169 m). Therefore, the reduction in leaf sizes could be explained by the effect of aridity and altitude on plants. In other words, it is a strategy in which species of the genus *Pistacia* react by reducing the transpiring surface when there is a lack of water (reducing the aridity index), thus coping with extreme living conditions at altitude (extreme cold, wind, dryness, intense UV light and radiation, low CO_2_ concentrations, etc. [13,14,15]). The same trend is reported in populations of *P. atlantica* located in high-altitude stations [6], and those in the most arid stations [8].

On the macromorphological level of the qualitative traits of the leaf, the results show that there is a large intraspecific divergence for the majority of the variables measured. The leaf wings are one of the most used characteristics for the taxonomic identification of species of the genus *Pistacia* [3]. In this study, almost all of the leaves show wider leaf wings on the rachis than the petiole (97.82%). The station Tgh, whose aridity index is equal to 0.18, has the lowest rate (82.43%) compared to the other stations. The leaf wings extend along the petiole in *P. lentiscus* and *P. atlantica* but the wings in *P. atlantica* are less developed than in *P. lentiscus* [4]. Regarding the color of the leaf, half of the leaves have a dark green color and the other half have a green color. This variable varies between dark green and green from one population to another. According to [12], leaflet color is one of the characteristics most affected by ecological factors. [16] explained this heterogeneity by the stationary parameters of the harvesting places (soil, climate, and slope). According to the same authors, the chlorophyll content tends to change its function in relation to the availability of water because water stress can cause the oxidation of chlorophyll pigments, resulting in the leaves having a pale green color. According to our results, all of the populations have petioles with a rounded shape and flattened on the adaxial face (100%).

Regarding the terminal leaflet, a total of 18.49% of the leaves of *P. lentiscus* have a TF, unlike other species of the genus *Pistacia* such as *P. atlantica*, *P. terebinhus*, and *P. vera* which have imparipinnate leaves. A mixture of paripinnate and odd-pinnate leaves in the same tree, such as lentisk, represents a natural variation in the same trait. For the shape of this leaflet, the narrow elliptical shape is the most frequent with variable rates of 19.73% (Saz), 17.12% (Tgh), 19.37% (Tiz), 17.56% (Berr), and 9.33% (Marss). For the apex of this leaflet, the mucronulate form is the most widespread at all stations with different proportions: Saz (15.05%), Tgh (14.41%), Tiz (19.03%), Berr (14.66%), and Marss (8.66%).

On the micromorphological level of the leaf, trichomes, waxes, and stomata are reliable traits for identifying species of the genus *Pistacia*. Indeed, the majority of the leaves are devoid of hairs, and when they are present, they are found with a low density. Unicellular and multicellular (bulb-shaped) peltate glandular trichomes, as well as non-glandular ciliated-type trichomes (sometimes branched), were observed under the SEM. [17] considers plants without furry hairs to be non-aromatic. According to [18], hairs are of paramount importance in systematics. As pointed out by several authors [3,4,19,20], the importance of trichomes, their presence, and their typology are used as taxonomic markers in the classification of pistachio trees. [21] noted that climate can have an effect on trichome morphology and number. In fact, in *Origanum vulgare*, Refs. [22,23] found that the number of glandular hairs decreases in species subjected to a continental climate. Ref. [24] showed that the density of covering (non-glandular) hairs increases with altitude, while glandular hairs decrease.

From another point of view, according to [25], no correlation could be demonstrated between hair types and habitat (degree of aridity) or geographical distribution. Hairiness, in general, acts as a screen that reflects sunlight [26]. This makes it possible to attenuate the absorption of UV radiation, minimize leaf overheating, and slow down the movement of air on the surface, which leads to a reduction in transpiration and constitutes an adaptation of the photosynthetic process to arid regions and deserts. Similarly, the dominance of glandular trichomes could be an adaptation to protect plants against large herbivores, insects [27,28], and the sandblasting effect in arid lands where winds are violent [29]. Some plants can tolerate high levels of metals, thanks to cysteine-rich defensive proteins [30] secreted in glandular trichomes, as shown in cadmium- and zinc-tolerant tobacco via their trichomes [31]. Some hairs could also play a role in water absorption in semi-desert habitats [25,32].

Stomata have also been proven to be an important characteristic from both taxonomic and evolutionary points of view [32]. The most common characteristic in the genus *Pistacia* was the occurrence of stomata only on the abaxial surface (hypostomatia). In this study, we recorded stomata along the principal nerve (principal rib). [33] classified *P. lentiscus* as hypostomatic and [34] suggested that this variation could be related to the ecological plasticity of *Pistacia* sp. to a wide range of environmental conditions. Indeed, our samples were collected on different sites with different climatic conditions (aridity and altitude). This demonstrates the remarkable plasticity of these species, which gives them the possibility of being able to survive in a very large geographical area of distribution. Similarly, a very significant variation was observed among the 11 provenances of *P. lentiscus* concerning this variable. It is higher on the abaxial face at ElHam (IA = 0.37; 420 st/mm^2^) and Sb (Alt. 1296 m; 388.6 st/mm^2^), on the other hand, the Tiz and Marss stations have the lowest values. For the adaxial face, the highest density values have been recorded at Saz (IA = 0.65) and Sb (Alt. 1296 m), while the Dar and Berr stations have the lowest values. It can be seen that the increase in the humidity of the environment is not necessarily accompanied by an increase in the number of stomata, as is the case for the two stations Tiz (IA = 0.5) and Marss (IA = 0.43). Al-Saghir (2005) suggested that amphistomaty is a characteristic related to the most primitive species, and hypostomacy or epistomacy is an evolutionary criterion of the *Pistacia* species. It is considered a strongly xeromorphic trait.

The loss of stomata can also be translated as an adaptation to climate change [34]. According to [35], amphistomaty could be an adaptation that facilitates a higher rate of photosynthesis in a sunny environment. [36] reported that the location of the stomatal apparatus on the underside of leaves with hairs are characteristics marking xerophytic adaptations. Resistant genotypes appear to be characterized by a low stomatal frequency and by small and distant stomata [37].

Regarding the size of the stomata on the abaxial side, an intra-specific variability was recorded. Indeed, the greatest values of the length and width of the stomata have been recorded at Dar (Alt. 1350 m), while the station Sb has the lowest values. Generally, they are sub-rounded. [9] reports rounded, sometimes elliptical, stomata for lentisk. [38] indicates that adaptation to drought involves a decrease in stomata size, while stomata density shows a more plastic response to environmental changes. In this context, [39] asserted that stomatal size is less influenced by exogenous factors than stomatal density.

Regarding the position of the stomata in the epidermis, there are little data available in the literature for species of the genus *Pistacia*. We cite the work of [5,6,7] for *P. atlantica* where the stomata are slightly sunken under the leaf epidermis. This agrees with our results for this species, as the stomata are at the same level to slightly sunken in the epidermis.

## 5. Conclusions

Our study reveals the existence of a very significant divergence of the studied characteristics between provenances and even between individuals. The genetic factor no doubt plays a key role in this variability, but it has been found that environmental factors also have a remarkable impact on the heterogeneity of the majority of leaf traits measured.

These results acquired on the macro- and micro-morphological variability of the leaf in *Pistacia lentiscus* showed a strong heterogeneity and even made it possible to understand their ability to adapt to extreme conditions of aridity and altitude. Indeed, all the populations studied have xeromorphic characteristics linked to leaves and stomata with degrees of adaptation differing from one population to another (size of leaves and stomata; density, shape, and position of stomata in the epidermis; types, structure, and epidermal hair density). There is an urgent need to conserve these resources and make the best use of them within the framework of projects for the rehabilitation of very degraded forest environments, such as the case of the green dam.

## Figures and Tables

**Table 1:** Ecological data from study stations.

Region	P (mm)	T° Min	T° Max	Station	GPS Coordinates	Altitude	IA
Djelfa	274.5	9.03	22.11	Dar chioukh (Dar)	34°56′1.58″ N	1350	0.18
					3°23′3.22″ E		
				Senalba (Sb)	34°38′40.91″ N	1296	0.2
					3°8′18.41″ N		
Medea	731	9.8	16.5	Berrouaghia (Berr)	36°10′36.18″ N	1169	0.39
					2°53′24.25″ E		
				El Omaria (Oma)	36°28′20.1″ N	779	0.35
					03°01′17.8″ E		
				Elhamdania (ElHam)	36°21′1.33″ N	398	0.37
					2°45′59.81″ E		
Boumerdes	613	12.2	21.56	Elkahla (Boum)	36°35′26″ N	495	0.45
					3°22′17″ E		
Tizi Ouzou	822	11.3	20.9	Taksebt (Tiz)	36°39′54.30″ N	209	0.5
					4° 6′58.86″ E		
Jijel	1020	11.1	19.6	Sidi-abdelaziz (Saz)	36°51′45.18″ N	24	0.65
					6° 4′21.59″ E		
Sidi Bel Abbès	347	8.5	20.6	Telagh (Tgh)	34°46′41.21″ N	963	0.18
					0°30′40.00″ E		
Marseille (France)	640	7.8	17	Montespin (Marss)	43°19′24.98″ N	216	0.43
					5°30′28.93″ E		

P: average annual rainfall, T° Max: average maximum temperatures for the warmest month, T° Min: average minimum temperatures for the coldest month, IA: aridity index (<0.03 = Hyper Arid, 0.03–0.2 = Arid, 0.2–0.5 = Semi-Arid, 0.5–0.65 = Dry Sub-humid, >0.65 = Humid) (source: CCN, WorldClim).

**Table 2:** Characteristics of the quantitative traits measured for the leaves of different populations of *Pistacia lentiscus*.

Character	Avg ± SD; Extent (C.V.)						
	*Pistacia lentiscus*						
	Leaf Length (cm)	Leaf Width (cm)	Number of Leaflets	Terminal Leaflet Length (cm)	Terminal Leaflet Width (cm)	The Length/Width Ratio of the Terminal Leaflet	Petiole Length (cm)
**Jijel (Saz)**	8.1 a*** ± 1.33 5.1–11.5 (16.4)	6.93 a*** ± 1.02 3.7–9.7 (17.2)	7.76 f*** ± 1.6 4–12 (21.07)	3.16 e*** ± 0.63 1.83–4.8 (20.4)	1.08 a*** ± 0.35 0.45–2 (32.67)	3.13 ab*** ± 0.86 1.64–4.87 (27.7)	1.54 d*** ± 0.3 0.82–2.5 (20.2)
**Tlagh (Tgh)**	6.53 c*** ± 1.1 4.5–9.7 (17.49)	4.4 e*** ± 1.01 2.6–8.5 (23.07)	8.8 abc*** ± 1.4 6–12 (16.78)	2.09 a*** ± 0.54 1.35–3.3 (25.7)	0.66 b*** ± 0.2 0.33–1.2 (37.5)	3.32 a*** ± 0.6 2.27–4.7 (20.3)	1.16 c*** ± 0.2 0.6–1.9 (19.34)
**Tizi Ouzou (Tiz)**	8.82 f*** ± 1.6 5–14.5 (18.83)	5.13 cd*** ± 1.1 2.2–8.6 (20.88)	9.8 d*** ± 1.61 5–15 (16.48)	2.7 cd*** ± 0.63 1.1–4 (23.57)	0.86 c*** ± 0.27 0.2–1.6 (32.41)	3.31 a*** ± 0.87 1.5–6 (26.26)	1.36 a*** ± 0.3 0.5–2.8 (27.85)
**Berrouaghia (Berr)**	7.31 e*** ± 1.3 3.2–13.5 (17.94)	4.69 b*** ± 0.6 1.3–7.3 (20.46)	8.49 ab*** ± 1.8 4–16 (21.99)	2.51 bc*** ± 0.5 1.2–4.1 (20.29)	0.83 c*** ± 0.28 0.2–2.3 (34.1)	3.24 ab*** ± 0.9 1.34–7.5 (28.02)	1.36 a*** ± 0.3 0.5–3 (23.6)
**Boumerdes (Boum)**	8.16 a*** ± 1.6 5.2–12.4 (19.6)	5.94 a*** ± 1.03 3.8–8.4 (17.46)	8.96 bc*** ± 1.4 6–12 (15.72)				1.28 ab*** ± 0.2 0.9–2 (19.18)
**Marseille (Marss)**	6.9 d*** ± 0.97 3.6–10.2 (14.09)	4.65 b*** ± 0.83 2.9–6.5 (17.9)	8.42 a*** ± 1.84 5–13 (21.88)	2.36 ab*** ± 0.65 1.2–3.5 (27.64)	0.65 b*** ± 0.26 0.2–1.1 (40.87)	3.93 c*** ± 1.19 2.45–7 (30.38)	1.2 bc*** ± 0.2 0.7–1.9 (20.88)
**Darchyoukh (Dar)**	9.9 b*** ± 0.85 7.8–12.1 (8.61)	5.27 d*** ± 0.82 3.5–10.1 (15.56)	10.32 e*** ± 2.03 4–16 (19.7)	2.31 ab*** ± 0.66 0.7–4.4 (28.82)	1.02 a*** ± 0.44 0.1–2.1 (43.59)	2.66 b*** ± 1.61 1.16–17 (60.55)	1.6 d*** ± 0.44 0.7–3.3 (27.3)
**El Omaria (Oma)**	9.61 b*** ± 1.48 5.7–13.1 (15.46)	4.93 c*** ± 0.9 2.8–7.7 (20.2)	10.4 e*** ± 1.7 6–14 (16.46)	2.85 d*** ± 0.7 0.6–3.9 (25.46)	1.08 a*** ± 0.44 0.1–2.6 (41.38)	2.99 ab*** ± 1.12 1.34–8 (37.38)	1.7 e*** ± 0.4 0.7–4 (28.02)
**ElHamdania (ElHam)**	8.3 a*** ± 1.06 5.6–11.2 (12.77)	6.1 a*** ± 0.7 4.6–9.3 (12.47)	9.2 c*** ± 1.31 6–12(14.29)				1.28 ab*** ± 0.2 0.9–2.2 (15.85)
**Senalba (Sb)**	10.3 g*** ± 1.01 7.9–12.8 (9.77)	7.4 f*** ± 0.7 5.9–10.2 (10.63)	9.6 d*** ± 1.5 6–12 (15.7)				2.23 f*** ± 0.4 1.2–3.5 (19.87)
**Average**	8.16 ± 1.73 3.2–14.5 (21.21)	5.09 ± 1.14 1.3–10.2 (22.42)	9.07 ± 1.96 4–16 (21.68)	2.58 ± 0.67 0.6–4.81 (26.24)	0.92 ± 0.38 0.1–2.6 (41.02)	3.1 ± 1.14 1.16–17 (36.91)	1.44 ± 0.41 0.5–4 (28.32)

**a**, **b**, **c**, **d**, **e**, **f**, **g** Separation of population groups by the Newman–Keuls test (*p* < 0.05). Values marked with the same letter are not significantly different. ***: significantly different. Average: Avg; Standard deviation: SD.; Extent: Min-Max; C.V.: Coefficient of variation (%).

**Table 3:** Factorial weights of the analyzed variables (correlations of the variables with the factors). Correlations ≥ 0.7 are in bold.

Variables	Factor 1	Factor 2
Leaf length (Ll)	**−0.75**	0.53
Leaf width (Lw)	**−0.80**	−0.17
Number of leaflet Pairs (Nl)	0.14	**0.84**
Terminal leaflet length (TLl)	**−0.70**	−0.53
Terminal leaflet width (TLw)	**−0.82**	−0.30
Terminal leaflet length/width Ratio (L/w TL)	0.55	0.07
Petiole length (Pl)	**−0.70**	0.13
Absence terminal leaflet (TL) %	0.37	−0.5

**Table 4:** Frequencies (%) for qualitative traits measured in different populations of *Pistacia lentiscus*.

Provenance		*P. lentiscus*					Average
		Jijel (Saz)	Tlagh (Tgh)	Tizi-Ouzou (Tiz)	Berrouaghia (Berr)	Marseille (Marss)	
Leaf rachis wing (Lrw)	0	0	0	0	0	0	0
	1	1	17.56	0	0	0	2.18
	2	98.99	82.43	100	100	100	97.82
Presence of terminal leaflet (TL)	0	78.93	82.88	79.58	81.33	89.33	81.51
	1	21.07	17.12	20.41	18.66	10.66	18.49
Size of terminal leaflet relative to basal leaflets (STL)	1	12.70	8.55	13.49	13.44	6	11.18
	2	8.36	5.4	2.07	5.22	0.66	4.89
	3	6.02	3.15	4.84	0	4	2.52
Shape of the terminal leaflet (ShTL)	1	0.66	0	1.03	0.23	0.66	0.43
	2	0	0	0	0	0	0
	3	0	0	0	0	0	0
	4	0	0	0	0	0	0
	5	0.66	0	0	0.78	0.66	0.53
	6	19.73	17.12	19.37	17.56	9.33	17.74
	99	0	0	0	0	0	0
Form of the apex of the terminal leaflet (FA)	1	15.05	14.41	19.03	14.66	8.66	14.89
	2	0	0	0	0.11	0.66	0.12
	3	0	0	0	0	0	0
	4	0	0	0	0	1.33	0.18
	5	2.34	2.7	0	0	0	0.69
	6	3.01	0	0	1.22	0	1.07
	7	0.33	0	0	2.22	0	1.12
	8	0	0	1.38	0.33	0	0.37
	9	0.33	0	0	0	0	0.06
Leaf margin (Lm)	1	100	100	100	100	100	100
	2	0	0	0	0	0	0
Form of the petiole (Fp)	1	0	0	0	0	0	0
	2	0	0	0	0	0	0
	3	100	100	100	100	100	100
Leaf color (Lc)	1	1	0.45	0	6.11	0	3.17
	2	25.75	99.54	58.47	32.11	60	45.48
	3	73.24	0	41.52	59.33	40	50.16
	99	0	0	0	2.44	0	1.18
TrichomeMargin	0	10.03	22.52	64.70	95	94.66	67.95
	1	89.63	77.02	35.3	5	5.33	31.93
	2	0.33	0.45	0	0	0	0.1
Trichome Midrib	0	19.06	13.51	70.58	72.33	91.33	58.01
	1	80.93	86.03	29.41	27.66	8.66	41.93
	2	0	0.45	0	0	0	0.05
TrichomeAdaxial F	0	23.07	15.76	88.93	69	93.33	60.32
	1	76.25	84.23	11.07	31	6.66	39.56
	2	0.66	0	0	36.33	0	0.1
Trichome Abaxial F.	0	18.06	11.71	94.46	59.22	90.66	54.94
	1	81.94	88.29	5.53	40.77	9.33	45.05
	2	0	0	0	0	0	0

**Table 5:** Characteristics of quantitative characteristics measured for stomata of different populations of *Pistacia lentiscus*.

Character	Avg ± SD; Extent (C.V.)			
	*Pistacia lentiscus*			
	Length of Stomata on the Abaxial Side (µm)	Width of Stomata on the Abaxial Side (µm)	Stomatal Density on the Abaxial Side (st/mm^2^)	Stomatal Density on the Adaxial Face (st/mm^2^)
Jijel (Saz)	27.91 c*** ± 3.22 20.08–37.72 (11.5)	21.73 de*** ± 2.97 12.6–28.86 (13.6)	320.66 a*** ± 68.69 125–541.66 (21.4)	17.5 f*** ± 25.99 0–125 (148.5)
Tlagh (Tgh)	27.05 a*** ± 2.77 19.1–35.3 (10.23)	22.09 e*** ± 2.39 14.66–27.5 (10.8)	345.83 b*** ± 79.58 166.6–583.3 (23.01)	2.66 abc*** ± 10.87 0–83.33 (407.97)
Berrouaghia (Berr)	25.93 b*** ± 3.17 15.49–39.44 (12.2)	20.8 b*** ± 3.04 14.4–37.74 (14.6)	313.7 a*** ± 80.22 125–558.3 (25.6)	0.76 ab*** ± 2.73 0–16.66 (357.15)
Dar chioukh (Dar)	29.46 g*** ± 4.15 12.7–42.67 (14.08)	23.27 h*** ± 2.76 14.98–31.09 (11.9)	308.53 a*** ± 76.98 125–508.3 (24.95)	0.53 a*** ± 2.41 0–16.66 (453.3)
Tizi Ouzou (Tiz)	26.14 b*** ± 2.47 17.49–31.21 (9.44)	19.71 a*** ± 2.23 13.22–29.15 (11.3)	166.4 c*** ± 55.57 75–375 (33.39)	6.3 d*** ± 7.39 0–25 (117.39)
Omaria (Om)	28.66 f*** ± 1.34 18.97–32.01 (4.69)	21.05 bc*** ± 1.45 16.70–24.55 (6.92)	353.5 b*** ± 91.04 100–591.6 (25.73)	4.33 cd*** ± 7.91 0–50 (182.5)
Marseille (Marss)	27.13 a*** ± 1.37 19.46–31.63 (5.07)	19.63 a*** ± 1.22 15.89–23.36 (6.22)	210.83 d*** ± 53.04 100–383.3 (25.16)	6.2 bd*** ± 7.79 0–25 (125.65)
Elhamdania (ElHam)	25.36 e*** ± 2.43 19.25–31.88 (9.61)	18.72 g*** ± 2.10 13.97–25.67 (11.22)	420.5 f*** ± 67.7 283.3–591.6 (16.1)	2.16 abc*** ± 4.35 0–25 (201.14)
Boumerdes (Boum)	27.44 ac*** ± 3.30 20.67–36.64 (12.04)	21.43 cd*** ± 2.57 15.52–29.79 (12.00)	339.7 b*** ± 53.68 175–483.3 (15.8)	3.16 bc*** ± 5.94 0–41.6 (187.8)
Senalba (Sb)	22.84 d*** ± 2.87 16.16–34.57 (12.59)	17.18 f*** ± 2.27 11.55–24.37 (13.21)	388.6 e*** ± 61.46 258.3–558.3 (15.8)	9.93 be*** ± 9.3 0–41.6 (93.7)
**Average**	26.88 ± 3.38 12.71–42.67 (12.6)	20.71 ± 2.96 11.55–37.74 (14.3)	319.05 ± 99.52 75–600 (31.2)	5.14 ± 11.27 0–125 (219.15

**a**, **b**, **c**, **d**, **e**, **f**, **g**, **h** Separation of population groups by the Newman–Keuls test (*p* < 0.05). Values marked with the same letter are not significantly different. ***: significantly different. Average: Avg; Standard deviation: SD.; Extent: Min-Max; C.V.: Coefficient of variation (%).

## Data Availability

Not applicable.

## Appendix A

**Table A1:** Qualitative variables measured for leaves.

Codification		Value
Leaf rachis wing (Lrw)	0	Absent
	1	Present in leaf rachis only
	2	Present in both leaf rachis and petiole
Presence of terminal leaflet (TL)	0	Absent (even pinnate leaf)
	1	Present (odd pinnate leaf)
Size of terminal leaflet relative to basal leaflets (STL)	1	Smaller than lateral ones
	2	As large as lateral ones
	3	Larger than lateral ones
Shape of the terminal leaflet (ShTL)	1	Lanceolat
	2	Ovate
	3	Ovate-oblong
	4	Oblone
	5	Elliptic
	6	Narrow elliptic
	99	Other
Form of the apex of the terminal leaflet (FA)	1	Mucronulate
	2	Acuminte
	3	Mucronate
	4	Caudate
	5	Cuspidate
	6	Acute
	7	Obtuse
	8	Retuse
	9	Emarginate
Leaf margin (Lm)	1	Leathery
	2	Membranaceous
Form of the petiole (Fp)	1	Flattznzd
	2	Rounded
	3	Rounded straight adaxially
Leaf color (Lc)	1	Light green
	2	Grenn
	3	Dark grenn
	99	Other
